# Integrative characterization of Huachansu against hepatocellular carcinoma: chemical profiling, Network pharmacology, and *in vivo* evidence of apoptosis and gut microbiota remodeling

**DOI:** 10.3389/fchem.2026.1782485

**Published:** 2026-05-13

**Authors:** Jinghui Zhang, Yang Chen, Zhuoling An, Hengyuan Yu

**Affiliations:** 1 Department of Pharmacy, Beijing Chao-Yang Hospital, Capital Medical University, Beijing, China; 2 Department of Clinical Pharmacy, Key Laboratory of Clinical Cancer Pharmacology and Toxicology Research of Zhejiang Province, Affiliated Hangzhou First People’s Hospital, School of Medicine, Westlake University, Hangzhou, Zhejiang, China; 3 School of Pharmacy, Hangzhou Normal University, Hangzhou, Zhejiang, China

**Keywords:** apoptosis, bufalin, cinobufotalin, hepatocellular carcinoma, Huachansu, molecular docking, network pharmacology

## Abstract

**Background:**

Hepatocellular carcinoma (HCC) remains a major global health burden, and traditional Chinese medicine (TCM) offers complementary therapeutic advantages. Although Huachansu (HCS), a clinically used TCM preparation, contains key bioactive components, including bufalin (BFL) and cinobufotalin (CBF), its antitumor mechanisms remain unclear.

**Methods:**

LC–MS profiling was performed to characterize the chemical constituents of HCS. Network pharmacology analysis was conducted by integrating predicted compound targets with HCC-associated genes to identify overlapping targets and enriched biological pathways. Subsequently, molecular docking was used to evaluate binding interactions between active compounds and hub proteins. Finally, the antitumor effects of BFL and CBF were validated *in vivo*, and gut microbiota alterations were assessed using 16S rRNA sequencing.

**Results:**

LC–MS profiling identified 17 HCS constituents, predominantly bufadienolides, establishing a chemical basis for mechanistic analyses. Network pharmacology analysis defined 142 overlapping targets enriched in apoptosis-related processes and cancer pathways. Molecular docking supported stable binding between bioactive compounds and key hub proteins, including STAT3, EGFR, AKT1, and PARP1, suggesting apoptosis-centered regulation. Guided by these *in silico* findings, *in vivo* experiments demonstrated that BFL and CBF reduced tumor burden and proliferation, as evidenced by hematoxylin and eosin staining and Ki-67 immunohistochemistry. Enhanced apoptosis was confirmed by TUNEL staining and Bax/Bcl-2 expression profiling, with more pronounced effects under combined treatment. Moreover, 16S rRNA sequencing revealed gut microbiota remodeling following BFL, CBF, and their combination treatments.

**Conclusion:**

These findings clarify the apoptosis-associated anti-HCC mechanism of HCS and provide mechanistic insights supporting development of TCM-based anticancer agents.

## 1 Introduction

Hepatocellular carcinoma (HCC) is among the most prevalent and lethal malignancies worldwide and imposes a substantial global health burden (Bray et al., 2024; Oh and Jun, 2023). Despite major advances in locoregional approaches and systemic regimens—including immune checkpoint inhibitor–based combinations and targeted therapies—clinical outcomes remain limited owing to tumor heterogeneity, treatment resistance, and therapy-associated toxicities (Gordan et al., 2024). These challenges highlight the need for complementary therapeutic strategies and deeper mechanistic insights that could expand the therapeutic landscape of HCC.

Traditional Chinese medicine (TCM) has long been used as an adjunct in cancer management and is often characterized by multicomponent, multitarget pharmacology (Yu et al., 2024a; Yu et al., 2024b). Huachansu (HCS), a clinically utilized TCM preparation derived from toad secretions, exhibits anticancer activity across multiple tumor types, including liver cancer (Huang et al., 2020; Wu Q. et al., 2024). Nevertheless, the precise bioactive constituents and the mechanistic basis underlying its effects in HCC remain insufficiently defined, limiting its rational optimization and mechanistic development. Chemically, HCS contains diverse classes of constituents, particularly bufadienolides and indole alkaloids, which are considered important material bases for pharmacological activity (Deng et al., 2024; Wu et al., 2025). Among them, bufalin (BFL) and cinobufotalin (CBF) are representative bufadienolides frequently associated with antitumor potential (Jiang et al., 2025; Wang J. et al., 2022; Wu S. et al., 2024). However, their key protein targets, pathway context, and integrated *in vivo* relevance in HCC have not been systematically characterized. Establishment of a mechanistic framework that connects compound–target interactions to tumor phenotypes is therefore essential.

Apoptosis dysregulation represents a hallmark of cancer and plays a pivotal role in hepatocarcinogenesis, tumor progression, and treatment response (Kroemer and Pouyssegur, 2008). Restoration of apoptotic signaling therefore remains a key objective in anticancer drug development (Kim et al., 2019). In the context of multicomponent therapeutics such as HCS, an apoptosis-centered mechanistic perspective may provide a tractable approach to linking predicted molecular targets with experimentally measurable antitumor effects.

In parallel, accumulating evidence highlights the importance of the gut–liver axis in HCC pathophysiology (Song et al., 2023). Microbial components and metabolites can reach the liver via portal circulation, influencing hepatic inflammation, immune regulation, and tumor-associated microenvironments. Alterations in gut microbiota composition have been associated with disease progression and therapeutic responses in liver cancer (Huang et al., 2025; Ren et al., 2025; Yadav and Kumar, 2025). However, whether key HCS-derived bioactive constituents modulate gut microbial communities in tumor-bearing hosts, and how such changes align with antitumor phenotypes, remains underexplored.

In this study, we aimed to establish an integrated framework to elucidate the anti-HCC mechanisms of HCS and its representative constituents. We first characterized the chemical profile of HCS using LC–MS to define its major constituents. We then applied network pharmacology analysis to identify candidate therapeutic targets and pathways relevant to HCC, followed by molecular docking to evaluate the binding feasibility between key compounds and hub proteins. Guided by these *in silico* analyses, we selected BFL and CBF for *in vivo* validation, with emphasis on apoptosis-related histological and molecular endpoints. Finally, we profiled gut microbiota alterations using 16S rRNA sequencing to characterize treatment-associated microbial community shifts in tumor-bearing mice. Together, this study provides a systematic framework for understanding HCS-derived anti-HCC activity through an apoptosis-centered mechanism while extending mechanistic exploration to the gut–liver axis.

## 2 Materials and methods

### 2.1 Chemicals and reagents

HCS capsules were obtained from Shandong Xinqi Pharmaceutical Co., Ltd. (Shandong, China, Lot No. [0010831472573]), with National Medical Products Administration (NMPA) execution standard Z20090944 (National Medical Products Administration, 2026). BFL and CBF were purchased from Chengdu Must Bio-technology Co., Ltd. (Sichuan, China). Pseudobufarenogin, arenobufagin, telocinobufagin, desacetylcinobufagin, bufotaline, cinobufagin, and resibufogenin were purchased from Chengdu Herbpurify Co., Ltd. (Sichuan, China). Gamabufotalin was purchased from Sichuan Vicky Biotechnology Co., Ltd. (Sichuan, China). These reference standards were used for LC–MS identification by comparison of retention times and MS/MS fragmentation patterns. LC–MS grade methanol, acetonitrile, and formic acid were purchased from Thermo Fisher Scientific (Waltham, MA, United States). Primary antibodies against Bax (AF0120) and Bcl-2 (AF6139) were purchased from Affinity Biosciences (Jiangsu, China). The TUNEL apoptosis detection kit was purchased from Thermo Fisher Scientific (Shanghai, China).

### 2.2 LC–MS-based chemical profiling of HCS

#### 2.2.1 Instruments

Sample extraction was performed using an ultrasonic extractor (KQ3200D, Kunshan Ultrasonic Instruments Co., Ltd., China). Centrifugation was conducted using a high-speed refrigerated centrifuge (Mikro 220R, Hettich Lab Technology, Germany). Chromatographic separation was performed on a Vanquish Flex UHPLC system (Thermo Fisher Scientific, Waltham, MA, United States) equipped with an ACQUITY UPLC HSS T3 reversed-phase column (2.1 mm × 100 mm, 1.8 μm; Waters Corporation, Milford, MA, United States). MS detection was performed on a Q Exactive hybrid quadrupole-Orbitrap mass spectrometer (Thermo Fisher Scientific).

#### 2.2.2 Sample preparation

Sample preparation was done by transferring 50 mg of the HCS capsule sample into a 2 mL centrifuge tube, followed by extraction with 1 mL of 80% methanol. The mixture was subjected to ultrasonication for 1 h and then centrifugation at 4 °C and 12,000 rpm for 10 min. Subsequently, 100 μL of the supernatant was diluted with 100 μL of ultrapure water, vortexed, and transferred into an injection vial for LC–MS analysis.

#### 2.2.3 Chromatographic conditions

Chromatographic separation was performed using solvent A (water containing 0.1% formic acid, v/v) and solvent B (acetonitrile). The flow rate was set to 0.3 mL/min, the column temperature was maintained at 40 °C, and the injection volume was 6.0 μL. The gradient elution program was as follows: 0–1.0 min, 98% A/2% B; 1.0–14.0 min, 98% A/2% B to 70% A/30% B; 14.0–25.0 min, 70% A/30% B to 0% A/100% B; 25.0–28.0 min, 0% A/100% B; 28.0–28.1 min, 0% A/100% B to 98% A/2% B; 28.1–29.5 min, 98% A/2% B.

#### 2.2.4 Mass spectrometry conditions

MS data were acquired on the Q Exactive system equipped with a HESI-II ion source operating in both positive and negative ionization modes. The spray voltages were set to 3.7 (positive mode) and 3.5 kV (negative mode). The capillary temperature was maintained at 320 °C and the auxiliary gas heater temperature was set to 300 °C. Sheath gas and auxiliary gas pressures were set to 30 psi and 10 psi, respectively. Nitrogen was used as the sheath, auxiliary, and collision gas (1.5 mTorr).

Data acquisition was performed in full scan/dd-MS^2^ mode. The full MS parameters were as follows: resolution 70,000 (at m/z 200), AGC target 1 × 10^6^, maximum injection time 50 ms, and scan range m/z 100–1500. The dd-MS^2^ parameters were as follows: resolution 17,500, AGC target 1 × 10^5^, maximum injection time 50 ms, isolation window 2.0 m/z, intensity threshold 1 × 10^5^, and up to 10 most intense precursor ions selected for fragmentation with dynamic exclusion. Collision energies were applied using stepped normalized collision energy values of 10, 30, and 60 eV. Base peak ion (BPI) chromatograms were used for visualization of the overall chemical profiles of HCS.

#### 2.2.5 Data processing and compound identification

Raw MS data were processed using Progenesis QI 3.0 (Waters) Following the standard workflow of raw data import, peak extraction, and deconvolution. Compound annotation was performed by querying the TCM Pro 2.0 database (Beijing Hexin Technology Co., Ltd.) and a self-constructed theoretical database compiled from published literature and public resources (Li et al., 2022; Wu et al., 2025). Final identification was determined through an integrated evaluation of retention time deviation (where reference substances were available), precursor mass error, MS/MS fragment matching, isotope distribution, and peak intensity/area.

### 2.3 Network pharmacology analysis

#### 2.3.1 Collection of HCS-related compounds and target prediction

Owing to incomplete coverage of Huachansu constituents in TCMSP, HERB, and BATMAN-TCM, HCS-related compounds were collected through literature mining in CNKI, ScienceDirect, and Web of Science and LC–MS profiling, yielding 44 candidate compounds. Molecular structures and SMILES strings were obtained from PubChem. Potential targets were predicted using SwissTargetPrediction and the Similarity Ensemble Approach platform. Compounds producing valid target predictions were retained, and duplicate targets were removed, yielding 13 active compounds and 499 putative targets for subsequent analysis.

#### 2.3.2 Identification of HCC-associated targets

HCC-associated genes were retrieved from GeneCards and the Online Mendelian Inheritance in Man database. GeneCards entries were filtered using a relevance score >1, and duplicate genes were removed to generate a final list of 1,441 HCC-related targets.

#### 2.3.3 Determination of overlapping targets between HCS and HCC

Predicted HCS targets (n = 499) and HCC-associated targets (n = 1,441) were imported into Venny 2.1.0 to obtain the intersection set. In total, 142 overlapping targets were identified and defined as putative therapeutic targets of HCS against HCC for downstream network and enrichment analyses.

#### 2.3.4 Construction of the compound–target network

To visualize the multicomponent and multitarget features of HCS, a compound–target network was constructed in Cytoscape. Network input files were prepared in Excel, where nodes represented HCS compounds and target genes/proteins and edges represented compound–target associations. Network visualization and basic topology analyses were performed using Cytoscape built-in tools.

#### 2.3.5 Protein–protein interaction network and hub target screening

The 142 overlapping targets were uploaded to the STRING database (species: *Homo sapiens*) to construct the protein–protein interaction (PPI) network. The minimum required confidence score was set to >0.4, while other parameters were retained as default. Isolated nodes were removed. The resulting PPI network was exported and analyzed in Cytoscape. Topological properties were evaluated using the NetworkAnalyzer tool, and degree centrality was used to identify highly connected nodes. The top 20 targets ranked by degree were defined as hub proteins.

#### 2.3.6 Gene Ontology (GO) and KEGG enrichment analyses

Functional enrichment analyses were performed to characterize the biological roles of the overlapping targets. The 142 targets were uploaded to the DAVID database with the identifier set to “OFFICIAL_GENE_SYMBOL” and species restricted to *H. sapiens*. GO enrichment was conducted for biological process, cellular component, and molecular function. KEGG pathway enrichment was conducted using the KEGG_PATHWAY category. Enrichment results were ranked by significance, and the top terms/pathways were selected for visualization.

### 2.4 Animals and ethics statement

All animal procedures were approved by the Hong Kong Special Administrative Region Department of Health (License number: (19-32) in DS/SHS/8/2/6 Pt.3) and conducted in accordance with institutional animal care guidelines. The tumor-bearing mouse study design was established as previously described (Zhang et al., 2021). Male BALB/c nude mice (4–6 weeks old) were housed under SPF conditions with standard husbandry. HepG2 cells (5 × 10^6^ cells in 100 μL PBS) were subcutaneously inoculated into the axillary region of each mouse. Following tumor establishment, mice were randomized into four groups (n = 8 per group): CON (saline), BFL (2 mg/kg), CBF (4 mg/kg), and BFL (2 mg/kg) + CBF (4 mg/kg) (Meng et al., 2009). Compounds were administered intraperitoneally once daily for 14 consecutive days. Tumor tissues and fecal samples were collected at the endpoint for downstream analyses, including 16S rRNA sequencing.

### 2.5 Hematoxylin and eosin (H&E) staining

Paraffin-embedded tumor tissues were sectioned using a rotary microtome (Leica RM2016), mounted onto glass slides, and oven-dried prior to staining. Sections were deparaffinized in xylene and rehydrated through graded ethanol to water. Following hematoxylin staining for 3–5 min, sections were differentiated in 1% hydrochloric acid solution, blued in 0.6%–0.7% ammonia water, and counterstained with eosin for 5 min. Sections were then dehydrated through graded ethanol, cleared in n-butanol and xylene, and mounted with neutral resin.

### 2.6 Immunohistochemistry

Paraffin sections were deparaffinized in xylene and rehydrated through graded ethanol to water. Antigen retrieval was performed in citrate buffer (pH 6.0) using microwave heating (medium 8 min to boiling, standing 8 min, and then medium–low 7 min), followed by cooling and PBS washes (3 × 5 min). Endogenous peroxidase activity was blocked with 3% H_2_O_2_ for 25 min at 25 °C in the dark. After blocking with 3% BSA for 30 min, sections were incubated with primary antibodies at 4 °C overnight, followed by HRP-conjugated secondary antibodies for 50 min at room temperature. DAB was used for chromogenic development under microscopic monitoring. Nuclei were counterstained with hematoxylin, differentiated, blued, dehydrated, cleared in xylene, and mounted.

### 2.7 TUNEL staining

Paraffin sections were deparaffinized in xylene and rehydrated through graded ethanol to water. Sections were treated with proteinase K at 37 °C for 25 min and then permeabilized at room temperature for 20 min, with PBS washes between steps (3 × 5 min). TdT enzyme and dUTP labeling solution were prepared at 1:9 and applied to the sections for incubation at 37 °C for 2 h in a humidified chamber. Endogenous peroxidase activity was blocked with 3% H_2_O_2_ for 15 min, followed by converter-POD incubation at 37 °C for 30 min. DAB development was monitored microscopically. Nuclei were counterstained with hematoxylin, and sections were dehydrated, cleared, and mounted.

## 3 Results

### 3.1 Identification of chemical constituents in the HCS extract

**FIGURE 1:**
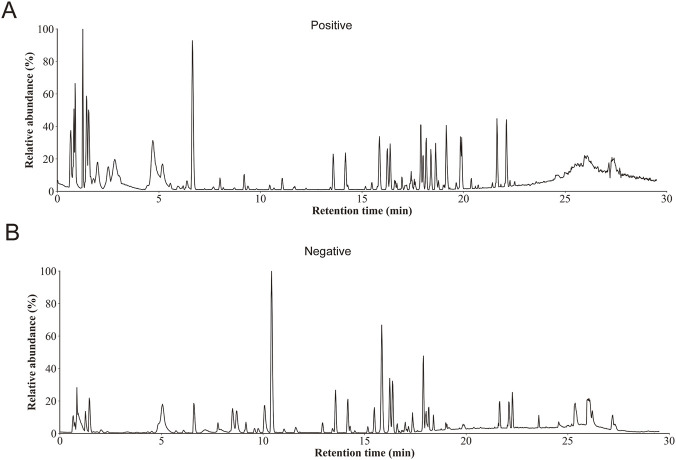
Base peak ion (BPI) chromatogram of Huachansu extract detected in positive **(A)** and negative **(B)** modes.

To better investigate the pharmacological effects of HCS, we first characterized its major chemical constituents using LC–MS analysis. The chemical profile of the HCS extract was acquired in both positive and negative ion modes, and the BPI chromatograms are shown in Figures 1A,B. BPI chromatograms displayed multiple well-resolved peaks distributed throughout the 0–30 min retention time window, indicating the presence of structurally diverse constituents in HCS.

Based on accurate mass measurements, retention times, adduct forms, and MS/MS fragmentation patterns, 17 compounds were identified (Table 1). These compounds mainly belonged to two major classes: indole alkaloids and bufadienolides. In the early elution phase, alkaloid components such as bufotenidine (Rt = 3.45 min) and dehydrobufotenine (Rt = 4.88 min) were detected, characterized by their relatively low molecular weights and distinct ionization behavior in the positive ion mode.

**TABLE 1:** The compound list of Huachansu (HCS) extract identified by LC-MS.

No.	Ion mode	Rt (min)	Adducts	Error (ppm)	Formula	Compound name	Fragments
1	POS	3.45	[M + H]^+^	−2.46	C_13_H_18_N_2_O	Bufotenidine	160.0754, 219.1489, 60.0812, 132.0803
2	POS	4.88	[M]^+^	−2.82	C_12_H_15_N_2_O+	Dehydrobufotenine	203.1176, 188.0943, 58.0656, 160.0754
3	NEG	13.58	[M + FA-H]^-^	−1.05	C_24_H_32_O_6_	Pseudobufarenogin	415.2129, 461.2183, 277.1443, 397.2012
4	POS	14.19	[M + H]^+^	−3.97	C_24_H_34_O_5_	Gamabufotalin	403.2486, 107.0853, 105.0699, 93.0699
5	NEG	15.85	[M + FA-H]^-^	−1.37	C_24_H_32_O_6_	Arenobufagin	461.2184, 415.2132, 397.2006, 353.2099
6	POS	16.25	[2M + H]^+^	−4.02	C_24_H_32_O_6_	Desacetylcinobufotalin	417.2264, 335.1999, 833.4462, 145.1010
7	POS	17.19	[M + H]^+^	−4.50	C_24_H_34_O_5_	Telocinobufagin	403.2473, 151.0388, 367.2260, 215.1797, 107.0854, 105.0698, 349.2153
8	POS	17.36	[M + H]^+^	−4.43	C_24_H_30_O_6_	Argentinogen	415.2111, 397.2008, 105.0698, 93.0698
9	POS	17.91	[M + H]^+^	−4.71	C_24_H_34_O_5_	Bufogenin B	403.2475, 402.2357, 349.2155, 367.2279
10	POS	18.02	[M + H]^+^	−4.79	C_24_H_32_O_5_	Desacetylcinobufagin	401.2308, 105.0696, 107.0852, 93.0697
11	POS	18.17	[M + H]^+^	−4.72	C_26_H_36_O_6_	Bufotaline	445.2578, 349.2156, 367.2260, 105.0698, 131.0854, 107.0853, 93.0698
12	POS	18.39	[M + H]^+^	−4.81	C_26_H_34_O_7_	Cinobufotalin	459.2366, 363.1945, 81.0697, 105.0699
13	NEG	18.86	[M + FA-H]^-^	−0.22	C_24_H_32_O_6_	Hellebrigenin	61.9883, 96.9599, 371.2222, 415.2132
14	POS	18.91	[M + H]^+^	0.26	C_24_H_34_O_4_	Bufalin	351.2307, 369.2444, 223.1459, 206.1241
15	POS	19.84	[M + H]^+^	−4.29	C_26_H_34_O_6_	Cinobufagin	443.2422, 105.0697, 187.1477, 107.0854, 151.0386, 365.2102, 93.0699
16	POS	20.37	[M + H]^+^	−4.71	C_24_H_32_O_4_	Resibufogenin	385.2374, 367.2256, 105.0696, 253.1947
17	POS	20.71	[M + H-2H_2_O]^+^	−4.09	C_24_H_34_O_6_	Hellebrigenol	383.2208, 81.0698, 105.0698, 93.0697, 107.0854, 119.0854, 145.1008

Most of the identified compounds were bufadienolides eluting between 13 and 21 min, including pseudobufarenogin, gamabufotalin, arenobufagin, telocinobufagin, argentinogen, bufogenin B, bufotaline, hellebrigenin, cinobufagin, and resibufogenin. Notably, CBF (Rt = 18.39 min, [M + H]^+^, C_26_H_34_O_7_) and BFL (Rt = 18.91 min [M + H]^+^, C_24_H_34_O_4_) were unambiguously identified with low mass errors, confirming their presence as representative bioactive constituents in HCS.

All MS data were processed using Progenesis QI 3.0. Compound identification was achieved through a comprehensive evaluation of retention time deviation, precursor mass accuracy, isotope distribution, and MS/MS fragmentation matching, supported by reference standards and theoretical databases constructed from published literature and public resources. These results provide a comprehensive chemical basis for subsequent pharmacological and mechanistic studies of HCS. The 17 constituents summarized in Table 1 represent high-confidence, representative compounds that were confirmed by comparison with reference standards under our LC–MS conditions, and therefore were reported as the standard-supported chemical basis for subsequent analyses.

### 3.2 Network pharmacology analysis identifies apoptosis-related targets of HCS in HCC

Based on the above-described chemical characterization of HCS, a network pharmacology strategy was employed to further explore its potential therapeutic targets in HCC. Because HCS is chemically complex and several commonly used TCM databases do not comprehensively index its constituents, we supplemented the LC-MS-confirmed compounds with additional literature-reported constituents to broaden chemical space coverage for hypothesis generation in network pharmacology. The LC-MS-confirmed compounds served as the experimental anchor, while literature-supported constituents were incorporated to improve representativeness of the multi-component features of HCS. By integrating experimentally identified constituents with literature-reported components, 13 representative HCS-related compounds were retained for subsequent target prediction, yielding 499 putative targets after duplicate removal.

**FIGURE 2:**
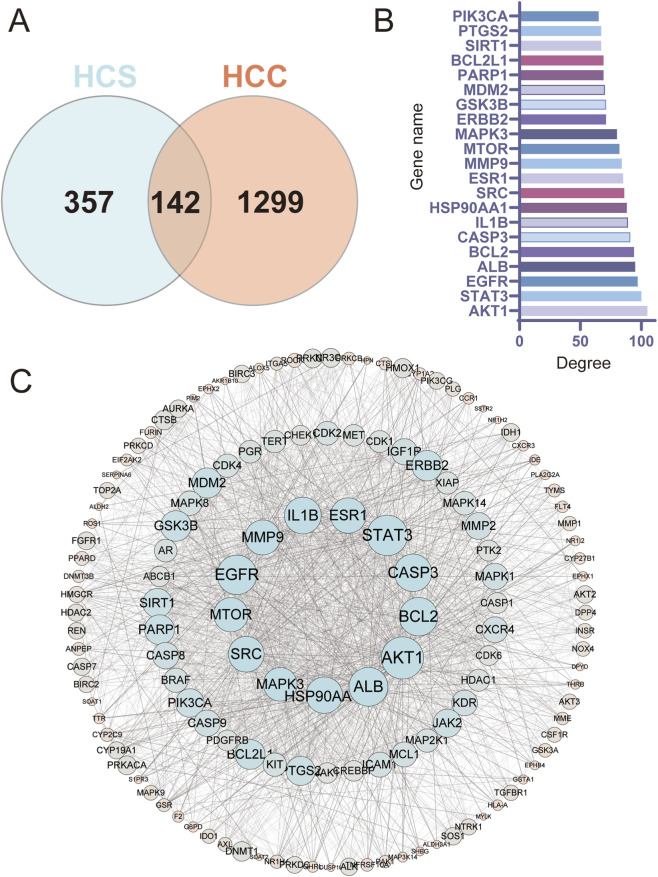
Network pharmacology analysis of HCS against hepatocellular carcinoma **(A)** Venn diagram showing the overlap between the predicted targets of HCS and HCC-related targets. In total, 142 common targets were identified. **(B)** Bar plot of the top 20 hub targets ranked by degree value based on protein–protein interaction network analysis. **(C)** PPI network of the overlapping targets between HCS and HCC. Node size reflects the degree value, and edges represent protein–protein interactions.

Meanwhile, 1,441 HCC-related targets were collected from disease databases. Intersection analysis revealed 142 common targets between HCS and HCC (Figure 2A), suggesting that these targets mediate the therapeutic effects of HCS against HCC.

**FIGURE 3:**
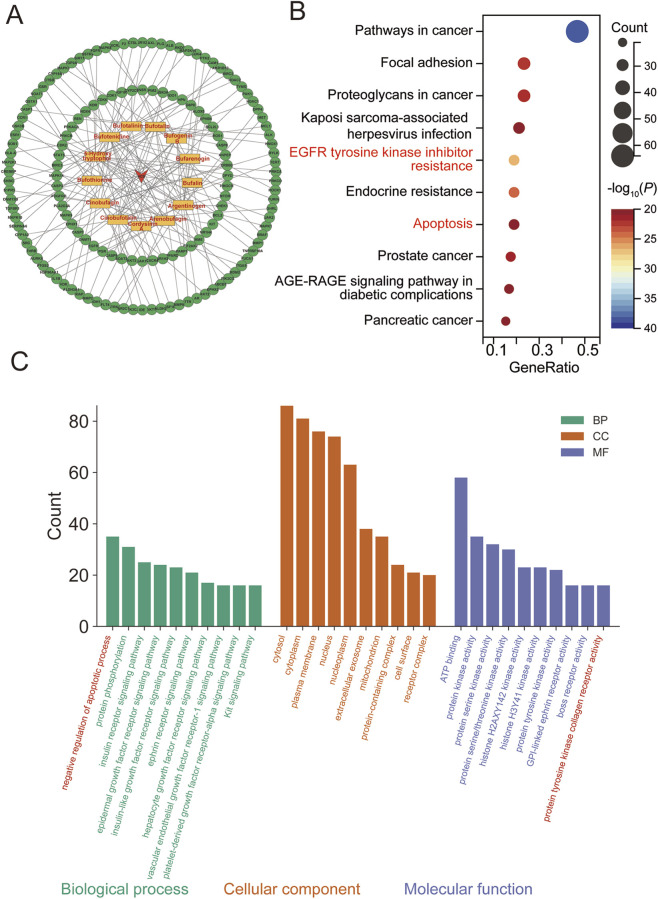
Compound–target network and functional enrichment analysis of HCS-related targets. **(A)** Compound–target network illustrating the interactions between active compounds of HCS and their corresponding HCC-related targets. Orange nodes represent active compounds, and green nodes represent target proteins. **(B)** KEGG pathway enrichment analysis of the overlapping targets. Dot size indicates the number of enriched genes, and color represents statistical significance (–log_10_ *P* value). **(C)** Gene Ontology (GO) enrichment analysis of the overlapping targets, including biological process, cellular component, and molecular function.

To further elucidate the interactions among these overlapping targets, a protein–protein interaction (PPI) network was constructed. Topological analysis identified 20 hub targets with the highest degree values (Figure 2B), including AKT1, STAT3, EGFR, CASP3, BCL2, mTOR, MMP9, ESR1, SRC, and HSP90AA1, many of which are closely associated with tumor progression and apoptosis regulation. The global PPI network further illustrated a dense interaction pattern centered on these hub nodes (Figure 2C), indicating their potential central roles in HCS-mediated anti-HCC activity. In parallel, a compound–target network was established to visualize the relationships between HCS constituents and their corresponding targets (Figure 3A). Multiple active compounds, including BFL and CBF, interacted with numerous apoptosis- and cancer-related targets, reflecting the multicomponent and multitarget characteristics of HCS.

Functional enrichment analysis was subsequently performed to clarify the biological relevance of the overlapping targets. KEGG pathway enrichment analysis revealed that the common targets were primarily enriched in cancer-related pathways, including pathways in cancer, Apoptosis, EGFR tyrosine kinase inhibitor resistance, Endocrine resistance, and Focal adhesion (Figure 3B). Notably, enrichment of the apoptosis pathway further supports the potential proapoptotic role of HCS in HCC treatment.

GO enrichment analysis demonstrated that these targets were significantly involved in biological processes related to apoptosis regulation, signal transduction, and protein phosphorylation. The enriched cellular components mainly included the cytosol, nucleus, plasma membrane, and protein-containing complexes. Molecular function analysis highlighted protein kinase activity, ATP binding, and protein tyrosine kinase activity (Figure 3C).

Collectively, these network pharmacology results suggest that HCS exerts its anti-HCC effects through coordinated regulation of multiple apoptosis-associated targets and signaling pathways. Key hub proteins identified in this analysis were selected for subsequent molecular docking studies to further evaluate compound–target interactions.

### 3.3 Molecular docking analysis validates the interactions between active compounds and hub targets

Based on the network pharmacology analysis, hub targets with high degree values were chosen to further evaluate their interactions with the corresponding active compounds of HCS through molecular docking. The crystal structures of the target proteins were retrieved from the Protein Data Bank, and docking simulations were performed using the AutoDock Vina algorithm.

As summarized in Table 2, multiple active compounds exhibited favorable binding affinities toward key apoptosis- and cancer-related targets. BFL showed strong binding to STAT3, with a Vina score of −8.2 kcal/mol, whereas bufarenogin demonstrated high affinity for EGFR (−8.9 kcal/mol) and HSP90AA1 (−7.1 kcal/mol). Notably, CBF exhibited strong binding interactions with several hub proteins, including GSK3β (−9.4 kcal/mol), PARP1 (−11.5 kcal/mol), MMP2 (−9.9 kcal/mol), and MMP9 (−7.3 kcal/mol), suggesting its potential as a key bioactive constituent of HCS.

**TABLE 2:** The vina score of docking.

No.	Target	Ligand	Vina score
1	AKT1	N-Lauryldiethanolamine	−5.2
2	STAT3	Bufalin	−8.2
3	EGFR	Bufarenogin	−8.9
4	HSP90AA1	Bufarenogin	−7.1
5	MMP9	Cinobufotalin	−7.3
6	ERBB2	N-acetyl-5-hydroxytryptamine	−49.1
7	GSK3β	Cinobufotalin	−9.4
8	PARP1	Cinobufotalin	−11.5
9	MMP2	Cinobufotalin	−9.9

**FIGURE 4:**
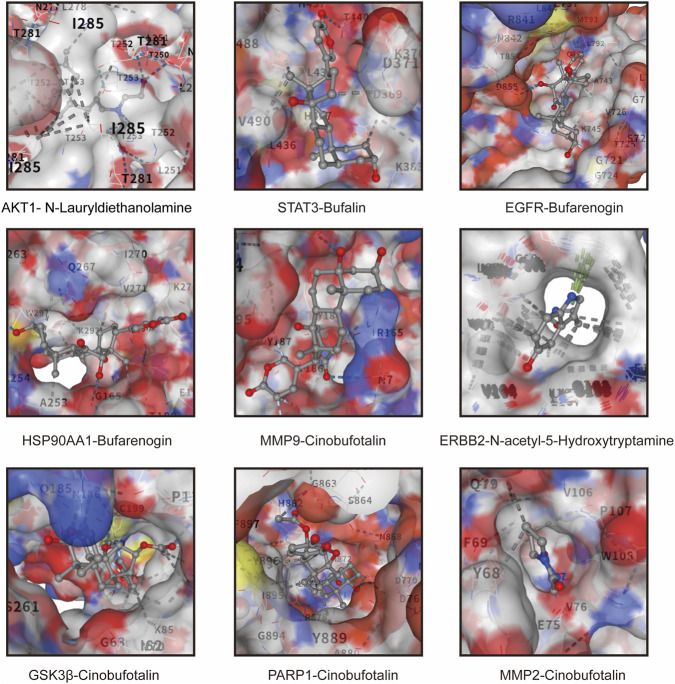
Molecular docking analysis of active compounds with hub targets. Representative docking conformations illustrating the predicted interactions between active compounds and key hub targets. The docking models depict interactions of AKT1 with N-lauryldiethanolamine, STAT3 with bufalin (BFL), EGFR with bufarenogin, HSP90AA1 with bufarenogin, MMP9 with cinobufotalin (CBF), ERBB2 with N-acetyl-5-hydroxytryptamine, GSK3β with CBF, PARP1 with CBF, and MMP2 with CBF. Target proteins are rendered as surface representations, while ligands are shown in stick models. Surface colors correspond to electrostatic surface potential, highlighting the predicted ligand-binding pockets and interaction regions.

Representative docking conformations further demonstrated the binding modes between active compounds and their respective targets. As shown in Figure 4, these compounds were stably accommodated within the predicted binding pockets through hydrogen bonding and hydrophobic interactions.

Overall, the molecular docking results support the multitarget binding potential of HCS-derived compounds and are consistent with the network pharmacology predictions, providing a structural rationale for subsequent experimental validation.

### 3.4 *In vivo* validation demonstrates the proapoptotic effects of BFL and CBF

Based on the molecular docking results, BFL and CBF were selected as representative active components of HCS for *in vivo* validation due to their strong predicted binding affinities toward apoptosis-related hub targets. Moreover, our prior experimental experience supported their robust antitumor activity (Zhang et al., 2020), and the key proteins highlighted by docking for these two compounds (e.g., STAT3 for BFL and PARP1 for CBF) are closely involved in apoptosis-related signaling (Wang X. et al., 2022; Zhong et al., 2024). To further support the docking result, molecular docking simulation indicated that the PARP1-CBF complex tended to stabilize during a 100 ns trajectory (Supplementary Figure S1). As network pharmacology and docking analyses collectively indicated apoptosis as a central mechanism underlying the anti-HCC effects of HCS, animal experiments were conducted to evaluate the effects of BFL, CBF, and their combination on tumor progression and apoptosis-associated pathways.

**FIGURE 5:**
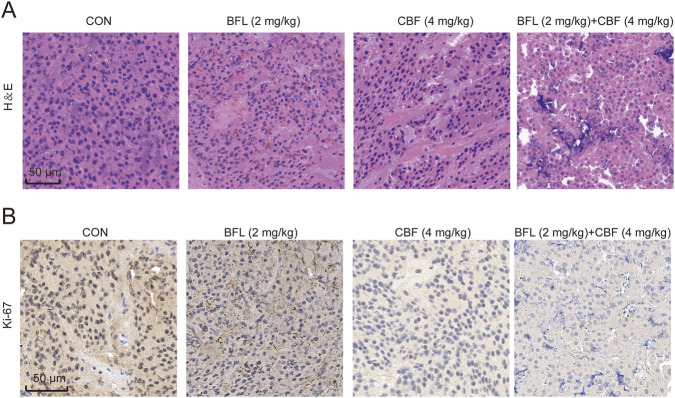
Effects of BFL and CBF on tumor histopathology and proliferation *in vivo*. **(A)** Representative hematoxylin and eosin (H&E)-stained tumor sections from the control (CON), BFL (2 mg/kg), CBF (4 mg/kg), and combination (BFL + CBF) groups. Treatment with BFL or CBF reduced tumor cell density, while the combination group exhibited the most pronounced histopathological changes. **(B)** Immunohistochemical (IHC) staining of Ki-67 in tumor tissues. Both BFL and CBF treatments decreased Ki-67 expression relative to the control group, with the combined treatment demonstrating the strongest anti-proliferative effect. Scale bar = 50 μm.

Histopathological evaluation using H&E staining revealed that tumor tissues from the control group exhibited dense tumor cell distribution (Figure 5A). In contrast, treatment with either BFL or CBF markedly reduced tumor cell density. The combination treatment group displayed the most pronounced histological alterations, characterized by extensive cellular loss and tissue damage.

To further assess tumor proliferative activity, Ki-67 immunohistochemical (IHC) staining was performed. As shown in Figure 5B, Ki-67 expression was significantly reduced in the BFL and CBF treatment groups compared with the control group, indicating suppressed tumor cell proliferation. Notably, the combined treatment resulted in the lowest Ki-67 expression, suggesting an enhanced anti-proliferative effect.

**FIGURE 6:**
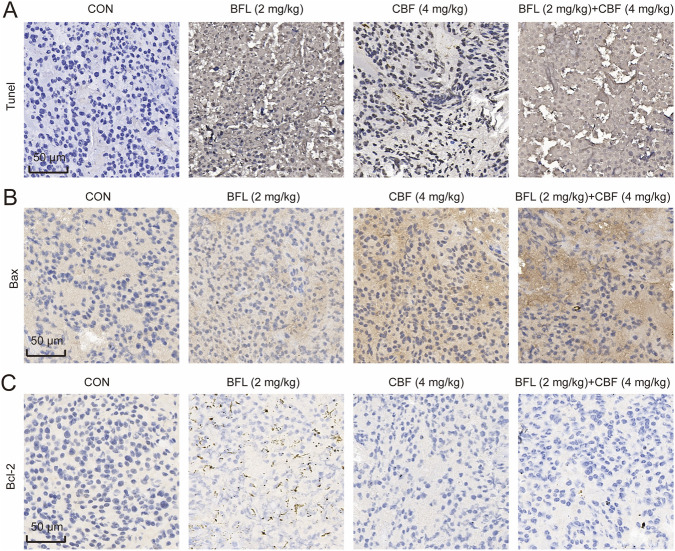
Effects of BFL and CBF on apoptosis in HCC tumor tissues. **(A)** TUNEL staining illustrating apoptotic cells in tumor tissues across different treatment groups. Both BFL and CBF increased apoptosis levels relative to the control group, with the combination group showing the strongest proapoptotic effect. **(B)** Immunohistochemical (IHC) staining of the proapoptotic protein Bax in tumor tissues. **(C)** IHC staining of the antiapoptotic protein Bcl-2 in tumor tissues. Treatment with BFL or CBF increased Bax expression and decreased Bcl-2 expression, whereas the combination group exhibited the most pronounced changes. Scale bar = 50 μm.

Apoptotic activity was examined using TUNEL staining. Both BFL and CBF treatments significantly increased the number of TUNEL-positive cells relative to the control group (Figure 6A), consistent with elevated apoptosis. This effect was further amplified in the combination group, indicating stronger apoptosis induction.

Given that several predicted hub targets function as upstream regulators of the mitochondrial apoptotic pathway, the expression of downstream apoptosis-related proteins was evaluated. IHC analysis demonstrated that the expression of the proapoptotic protein Bax was markedly upregulated, whereas that of the anti-apoptotic protein Bcl-2 was downregulated following BFL or CBF treatment (Figures 6B,C). These changes were most pronounced in the combined treatment group, further supporting the enhanced proapoptotic effects of BFL and CBF *in vivo*.

Collectively, these findings demonstrate that BFL and CBF inhibit tumor progression and promote apoptosis in HCC models, with combined administration producing superior therapeutic effects. These results provide experimental validation for the apoptosis-mediated antitumor mechanism of HCS predicted by the network pharmacology and molecular docking analyses.

### 3.5 Gut microbiota profiling reveals community shifts induced by BFL and CBF

Given the close interplay between the gut–liver axis and HCC progression, as well as emerging evidence that gut microbiota can influence host responses to pharmacological interventions, we investigated whether BFL and CBF, administered alone or in combination, were associated with alterations in the gut microbial community of tumor-bearing mice using 16S rRNA sequencing.

**FIGURE 7:**
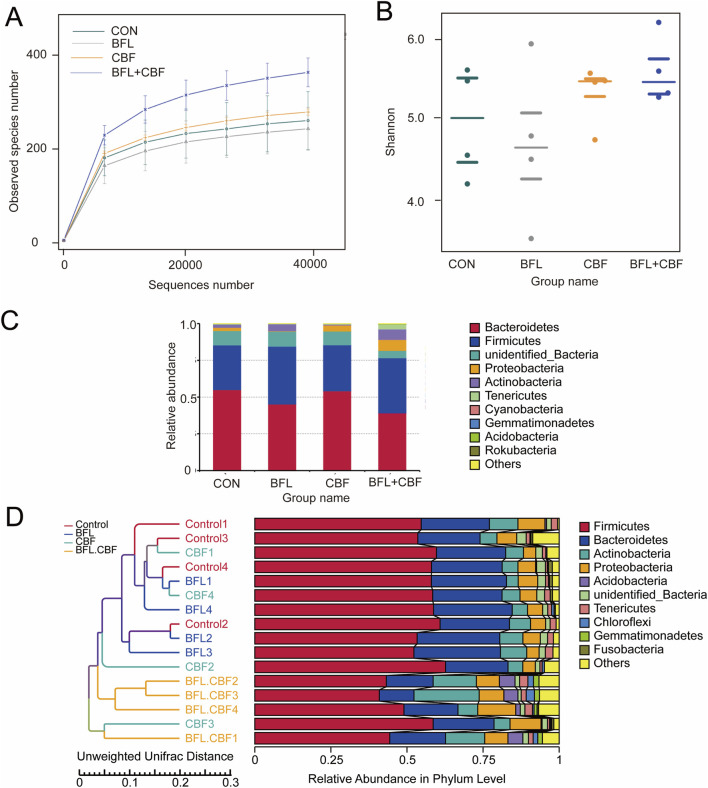
Gut microbiota alterations in tumor-bearing mice following BFL and CBF interventions. **(A)** Rarefaction curves based on the observed species index for all samples from the CON, BFL, CBF, and BFL + CBF groups, indicating adequate sequencing depth. **(B)** Alpha (α) diversity of the gut microbiota across groups assessed using the Shannon diversity index. **(C)** Phylum-level taxonomic composition showing the relative abundances of bacterial taxa in each group. **(D)** Hierarchical clustering of samples based on unweighted UniFrac distances derived from OTU profiles, with corresponding phylum-level relative abundance profiles displayed alongside.

Rarefaction curves based on the observed species index approached a plateau with increasing sequencing depth (Figure 7A), indicating adequate sequencing coverage across all groups. Alpha diversity was evaluated using the Shannon index. Compared with the control group, both the BFL and CBF treatment groups exhibited shifts in Shannon diversity, whereas the BFL + CBF group displayed a more pronounced increase (Figure 7B), suggesting that combined administration was associated with stronger modulation of within-sample microbial diversity.

At the phylum level, stacked bar plots showed that Bacteroidetes and Firmicutes were the dominant taxa across samples (Figure 7C). Notably, the BFL + CBF group showed an increased relative abundance of Firmicutes accompanied by a reduction in Bacteroidetes, indicating a more evident compositional shift at the community level.

To assess between-sample differences, hierarchical clustering based on unweighted UniFrac distances was performed. The clustering pattern indicated treatment-associated restructuring of the gut microbiota, with the BFL + CBF group exhibiting clearer separation from the control and single-treatment groups (Figure 7D). Collectively, these findings suggest that BFL and CBF are associated with gut microbiota remodeling in tumor-bearing mice, with their administration producing more pronounced community-level shifts. These observations provide a basis for subsequent analyses aimed at identifying key differential taxa and microbiota-related features linked to the observed host responses.

## 4 Discussion

In this study, we combined LC–MS profiling, network pharmacology, molecular docking, and *in vivo* experiments to investigate the anti-HCC mechanism of HCS. LC–MS identified 17 representative constituents dominated by bufadienolides, and network analysis suggested an apoptosis-related mechanism. Guided by docking, BFL and CBF were prioritized for validation. In tumor-bearing mice, treatment reduced tumor cell density and Ki-67 expression and consistently enhanced apoptosis, reflected by increased TUNEL positivity, higher Bax, and lower Bcl-2. The combined regimen produced stronger effects than either monotherapy, while formal synergy evaluation will require dedicated quantitative assessment.

Beyond tumor-intrinsic mechanisms, microbiota analysis introduces an additional dimension relevant to the gut–liver axis, which is increasingly recognized as a key modulator of liver cancer biology and therapeutic responsiveness (Yadav and Kumar, 2025). Our results revealed a concurrent upregulation of the pro-apoptotic Bax/Bcl-2 ratio and a distinct structural shift in the gut microbiota, characterized by an enrichment of Firmicutes and a reduction in Bacteroidetes following BFL + CBF treatment. This compositional remodeling, particularly the increased relative abundance of Firmicutes, is of profound biological significance. The Firmicutes phylum encompasses a wide array of beneficial bacteria recognized as the primary producers of SCFAs (Koh et al., 2016). Through the highly interconnected gut-liver axis, microbiota-derived SCFAs translocate into the hepatic microenvironment, where they exert potent indirect anti-tumor effects. Mechanistically, SCFAs have been shown to function as endogenous histone deacetylase inhibitors, which effectively reverse the Warburg effect and induce tumor cell apoptosis by upregulating the Bax/Bcl-2 ratio and activating the caspase cascade (Feitelson et al., 2023; Mirzaei et al., 2021). However, the microbiota findings in this study remain associative. Establishing causality will require well-controlled microbiome perturbation and transfer experiments (for example, antibiotic-mediated depletion and fecal microbiota transplantation), coupled with targeted metabolomic profiling to quantify relevant microbial metabolites such as SCFAs and bile acids (Kim et al., 2024; Metwaly et al., 2025).

Additionally, several limitations should be acknowledged. Although our integrated workflow combining network pharmacology, molecular docking, and *in vivo* validation supports an apoptosis-centered anti-HCC activity of HCS, this strategy inevitably depends on the completeness of public databases and the predictive performance of computational algorithms. Therefore, direct biochemical or cell-based assays will be necessary to establish causal relationships between prioritized compounds and their proposed molecular targets. In future studies, SPR or thermal shift analysis can be included to experimentally confirm direct binding, particularly for PARP1. Because ionization efficiency varies among compounds, LC–MS acquired under a single set of conditions may not capture all HCS constituents, and additional components reported in previous studies may be missed (Deng et al., 2024). Moreover, the present LC–MS analysis was used for qualitative identification and does not provide robust relative amounts or batch-to-batch variation of individual constituents; targeted quantitative assays with suitable standards will be needed for comprehensive quantification. Moreover, the experimental validation focused on two representative and highly prioritized constituents, BFL and CBF, selected on the basis of *in silico* analyses. Although BFL and CBF share a bufadienolide core, their different substitution patterns may affect polarity and binding-site complementarity, potentially resulting in partially distinct target engagement. This is consistent with our network pharmacology and docking results showing overlapping but non-identical target profiles, which may broaden pathway coverage under combination treatment. However, HCS comprises multiple additional constituents that may contribute to its anti-HCC efficacy, either through independent mechanisms or cooperative interactions. Systematic characterization of these components and their potential combinatorial effects remains an important objective for future studies. Finally, the more pronounced phenotypic effects observed under combined BFL and CBF treatment warrant further investigation within clinically relevant frameworks, including dose optimization, pharmacokinetic profiling, safety evaluation, and rigorous quantitative assessment of additivity or synergy. Because pharmacokinetic properties were not assessed here, future studies incorporating PK and exposure–response analyses are needed to determine whether the combination benefit is partly driven by improved bioavailability or tissue exposure.

In summary, our findings support a model in which HCS-derived bufadienolides, represented by BFL and CBF, suppress HCC predominantly through apoptosis activation. This interpretation is supported by systems-level target prediction, docking-based plausibility, and consistent *in vivo* evidence. The antitumor effects are accompanied by gut microbiota remodeling, reflected in community-level structural shifts and changes in the Firmicutes-to-Bacteroidetes ratio. Collectively, this integrated framework provides a rational starting point for further mechanistic dissection and optimization of HCS-based interventions, while motivating future studies aimed at establishing causal links between microbiota metabolites and apoptosis-associated tumor regulation along the gut–liver axis.

## 5 Conclusion

In conclusion, this study provides an integrated, multilevel characterization of the anti-HCC effects of HCS. LC-MS profiling established the major chemical composition of HCS and identified BFL and CBF as representative bufadienolides. Network pharmacology analysis revealed 142 overlapping HCS-HCC targets enriched in apoptosis-related functions and cancer-associated pathways, while molecular docking supported stable interactions between key ligands and hub proteins, including STAT3, EGFR, AKT1, and PARP1, collectively indicating an apoptosis-centered regulatory framework. Experimental validation *in vivo* demonstrated that BFL and CBF suppressed tumor burden and proliferation (H&E and Ki-67) and promoted apoptotic responses (increased TUNEL positivity, Bax upregulation, and Bcl-2 downregulation). Notably, combined treatment produced more pronounced biological effects. In parallel, 16S rRNA sequencing indicated that BFL and CBF were associated with gut microbiota remodeling, implying that microbiota community shifts may accompany the observed antitumor responses. Overall, these findings clarify key bioactive constituents and support an apoptosis-associated mechanism underlying HCS activity in HCC. They provide a practical framework for the mechanistic exploration and optimization of TCM-derived anticancer strategies.

## Data Availability

The original contributions presented in the study are publicly available. This data can be found here: https://doi.org/10.6084/m9.figshare.32188962.
