# Supplementary material for: Integrative characterization of Huachansu against hepatocellular carcinoma: chemical profiling, Network pharmacology, and in vivo evidence of apoptosis and gut microbiota remodeling
Source: Front Chem. 2026 May 13;14:1782485. doi: 10.3389/fchem.2026.1782485 (PMC13212497; doi:10.3389/fchem.2026.1782485)
Supplement: Supplementary file 1 [file DataSheet1.docx]

**Supporting Information for**

**Integrative Characterization of Huachansu Against Hepatocellular Carcinoma: Chemical Profiling, Ne twork Pharmacology, and In Vivo Evidence of Apoptosis and Gut Microbiota Remodeling**

Jinghui Zhang^1^, Yang Chen^2^, Zhuoling An^1,*^, Hengyuan Yu^3,*^

^1^ Department of Pharmacy, Beijing Chao-Yang Hospital, Capital Medical University, Beijing, China.

^2^ Department of Clinical Pharmacy, Key Laboratory of Clinical Cancer Pharmacology and Toxicology Research of Zhejiang Province, Affiliated Hangzhou First People's Hospital, School of Medicine, Westlake University, Hangzhou, Zhejiang 310006, China;

^3^ School of Pharmacy, Hangzhou Normal University, Hangzhou, Zhejiang 311121, China.

Table S1. Representative Huachansu (HCS)-related compounds used for target prediction (n = 13)

| **No.** | **Compound name** |
| --- | --- |
| 1 | Bufotenidine |
| 2 | Cyclo(Pro–Gly) |
| 3 | N-Lauryldiethanolamine |
| 4 | Bufogenin B |
| 5 | Bufarenogin |
| 6 | Arenobufagin |
| 7 | N-acetyl-5-Hydroxytryptamine |
| 8 | Cinobufagin |
| 9 | 5-Hydroxy tryptophol |
| 10 | Bufotalin |
| 11 | Argentinogen |
| 12 | Bufalin |
| 13 | Cinobufotalin |

Table S2. The vina score of docking between supplementary targets and ligands

| **No.** | **Target** | **Ligand** | **Vina score** |
| --- | --- | --- | --- |
| 1 | SIRT1 | Cyclo(pro-gly) | -5.3 |
| 2 | PIK3CA | Cinobufotalin | -10.3 |
| 3 | JAK2 | Cinobufotalin | -10.3 |
| 4 | CASP3 | Bufotenidine | -5.3 |
| 5 | IL1B | Bufalin | -8.9 |


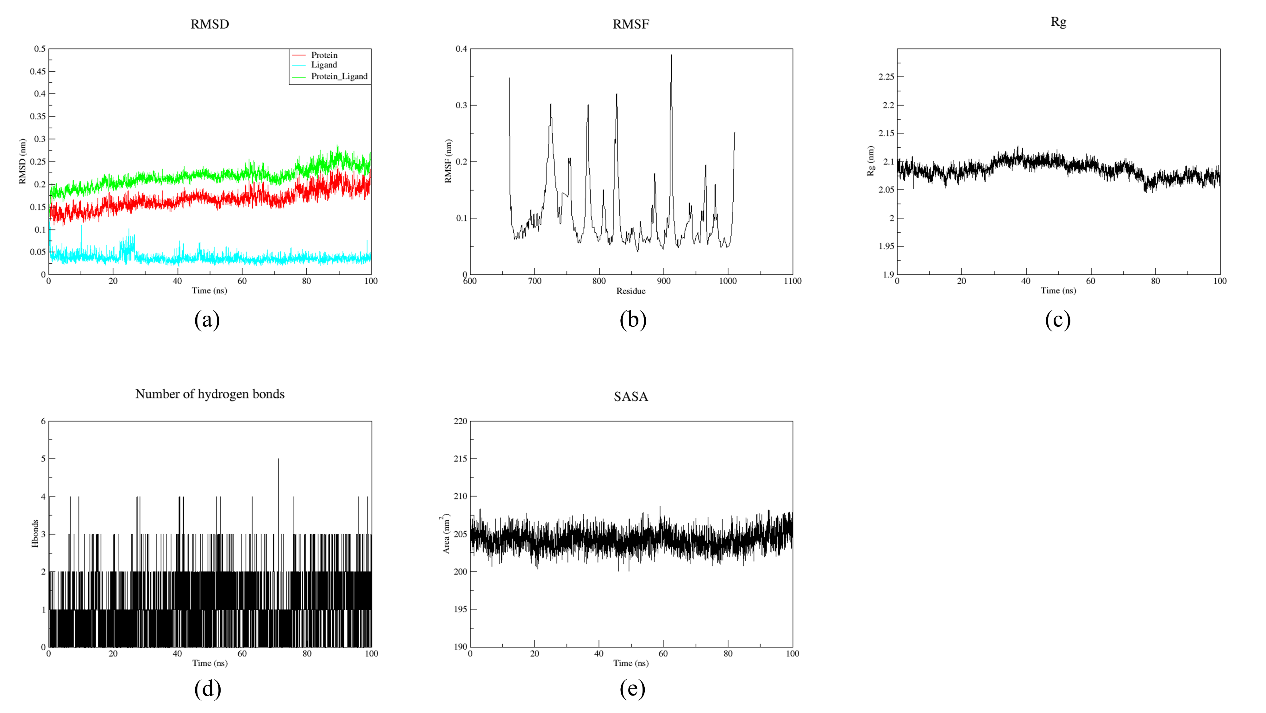


**Figure S1**. Analysis of molecular dynamics simulation results. (a) Root-mean-square deviation (RMSD) of the protein and ligand. (b) Root-mean-square fluctuation (RMSF) of protein residues. (c) Radius of gyration (Rg) of the protein during the simulation. (d) Time-dependent changes in the number of hydrogen bonds (H-bonds). (e) Solvent-accessible surface area (SASA) of the protein.





**Figure S2.** Pilot study on the effects of bufalin (BFL) and cinobufotalin (CBF) on tumor growth curves.
